# Study on the mechanisms of action of berberine combined with fluconazole against fluconazole-resistant strains of *Talaromyces marneffei*

**DOI:** 10.3389/fmicb.2022.1033211

**Published:** 2022-11-14

**Authors:** Pan Kai-su, Luo Hong, Zheng Dong-yan, Zheng Yan-qing, Alex Andrianopoulos, Jean-Paul Latgé, Cao Cun-wei

**Affiliations:** ^1^Department of Dermatology and Venereology, The First Affiliated Hospital of Guangxi Medical University, Nanning, Guangxi, China; ^2^Guangxi Key Laboratory of Mycosis Research and Prevention, Nanning, China; ^3^Department of Dermatology, Changsha First Hospital, Changsha, China; ^4^Fourth People’s Hospital of Nanning, Nanning, China; ^5^School of Biosciences, The University of Melbourne, Parkville, VIC, Australia; ^6^Institute of Molecular Biology and Biotechnology, FORTH and School of Medicine, University of Crete, Crete, Greece

**Keywords:** *Talaromyces marneffei*, berberine, fluconazole, fluconazole-resistant, combination therapy

## Abstract

*Talaromyces (Penicillium) marneffei* (*T. marneffei*) is a thermally dimorphic fungus that can cause opportunistic systemic mycoses. Our previous study demonstrated that concomitant use of berberine (BBR) and fluconazole (FLC) showed a synergistic action against FLC-resistant *T. marneffei* (B4) *in vitro*. In this paper, we tried to figure out the antifungal mechanisms of BBR and FLC in *T. marneffei* FLC-resistant. In the microdilution test, the minimum inhibitory concentration (MIC) of FLC was 256 μg/ml before FLC and BBR combination, and was 8 μg/ml after combination, the partial inhibitory concentration index (FICI) of B4 was 0.28. After the treatments of BBR and FLC, the studies revealed that (i) increase reactive oxygen species (ROS), (ii) reduce ergosterol content, (iii) destroy the integrity of cell wall and membrane, (iv) decrease the expression of genes *AtrF*, *MDR1*, *PMFCZ*, and *Cyp51B* however *ABC1* and *MFS* change are not obvious. These results confirmed that BBR has antifungal effect on *T. marneffei*, and the combination with FLC can restore the susceptibility of FLC-resistant strains to FLC, and the reduction of ergosterol content and the down-regulation of gene expression of *AtrF, Mdr1, PMFCZ*, and *Cyp51B* are the mechanisms of the antifungal effect after the combination, which provides a theoretical basis for the application of BBR in the treatment of Talaromycosis and opens up new ideas for treatment of Talaromycosis.

## Introduction

Talaromycosis is a characteristic opportunistic infection of HIV/AIDS patients in endemic areas. It is a serious disease with a mortality rate of up to 93% due to the insufficient efficacy of the drug used (Hu et al., 2013). Currently, this disease is treated according to the guidelines and is based on amphotericin B, or itraconazole or voriconazole, FLC as a low-cost and relatively safe drug, is not included in the guidelines because of its poor therapeutic effect in *Talaromyces marneffei* (Hoenigl et al., 2021*)*. Traditional Chinese medicine used alone or in combination (Cao et al., 2009; Mo et al., 2014) may be useful to set up more effective therapy against this fungal infection. Most of the known fungal efflux pumps conferring azole resistance are ABC transporters(Leppert et al., 1990).The clarified target of FLC is the lanosterol 14α demethylase, a key enzyme responsible for the synthesis of ergosterol which is a pivotal component in cell membrane encoded by *ERG11* (Zavrel and White, 2015*)*, respiration deficiency leading to decreased reactive oxygen species (ROS) and up-regulation of drug efflux pump mediated by ATP-binding cassette superfamily (APC transporter) and Mdr1p, a member of major facilitator superfamily (MFS) have also been recently documented to result in antifungal resistance to FLC (Patrick et al., 2005). *AtrF* and *Cyp51B* played an important role in the triazole resistance *Aspergillus fumigatus* strains (Yu et al., 2021). BBR is an alkaloid that has long been used to treat bacterial gastroenteritis and dysentery. In recent years, other pharmacological effects of BBR have been gradually discovered, of which the synergistic antifungal effects against FLC-resistant *Candida albicans*, *Candida tropicalis*, and *Cryptococcus* in combination with FLC are receiving increasing attention, *via* ROS increase, intracellular drug accumulation, ergosterol decrease and efflux inhibition (Xu et al., 2009; Bang et al., 2014; Dhamgaye et al., 2014; Shi et al., 2017). Previous combination drug sensitivity test of BBR against *T. marneffei* have suggested that BBR not only has potent antifungal effects against *T. marneffei* by itself, but also exhibits good synergistic effects in combination with FLC *in vitro*, especially for FLC-resistant strains showed synergistic effect (Luo et al., 2019). The aim of this study was to elucidate the synergistic mechanisms of the combined of BBR and FLC in FLC-resistant *T. marneffei*.

## Materials and methods

### Isolates, cultivation, and agent

The *T. marneffei* wide type strain FRR 2161 (WT) was kindly provided by Prof. Alex Andrianopoulos from the School of Biosciences, The University of Melbourne, Australia. The *T. marneffei* FLC-induced resistant strain B4 (point mutation G1587T, amino acid substitution G441V) was obtained in our laboratory by *in vitro* induction from the *T. marneffei* standard strain FRR 2161, and resistance was verified not to diminish or disappear over generations. *T. marneffei* strains were inoculated into brain-heart infusion (BHI) agar medium at 37°C and cultured for transmission. After microscopic observation, 95% or more of the cells were found to be in the yeast phase and then used for the experiment. The yeast cells were collected and the cell concentration was adjusted by turbidimetric method. The cell concentration was adjusted with the turbidimeter and diluted to 1–5 × 10^3^ CFU/ml. Fluconazole (FLC, Pfizer Inc., Madrid, Spain) was obtained as pure powder and diluted in sterile distilled water, Berberine (BBR, Sigma–Aldrich, St Louis, MO, USA) was prepared in dimethyl sulfoxide (DMSO). Stock solutions were diluted in RPMI 1640 medium (Sigma Chemical Co., St. Louis, Mo.) and then serially diluted fourfold to achieve the final strength required for the test.

### *In vitro* antifungal activity

Antifungal susceptibility testing was performed using the checkerboard broth microdilution method according to CLSI protocol M27-A3 (Clinical and Laboratory Standards Institute) with some modifications: The final concentration of FLC was set at 64 μg/ml in the range of 0.125–64 μg/ml for WT and 1,024 μg/ml in the range of 2–1024 μg/ml for B4 and BBR. Antifungal plates were incubated at 37°C and MIC assays were read 72 h after inoculation. The susceptibility test was repeated three times at different times using *Candida parapsilosis* ATCC 22019 and *Candida krusei* ATCC 6458 as quality control strains. The fractional inhibitory concentration index (FICI) was used to classify drug interaction. The FICI = MIC (A combo)/MIC (A alone) + MIC (B combo)/MIC (B alone). Synergy and antagonism were defined by FICI of ≤0.5 and﹥4, respectively. An FICI result of﹥0.5 but ≤4 was considered indifferent.

### Growth curve assay

Exponentially growing yeast cells were harvested and resuspended in fresh yeast extract-peptone dextrose medium (YPD) to obtain a final concentration of 1 × 10^5^ CFU/ml. Different concentrations of BBR and ergosterol (alone or mixed) were added to the cells. Cells were incubated under shaking 200 rpm at 37°C, and OD600 was measured at the indicated time points after incubation (0, 4, 8, 12, 24, 36, and 48 h). The same volumes of solvents (DMSO, Tween 80. and ethanol) were added to the untreated controls. Three independent experiments were performed at three different time points for optimal results.

### Transmission electron microscopy

RPMI1640 medium was used to collect 2 × 10^6^ CFU/ml of yeast cells. Then, 32 μg/ml BBR, 2 μg/ml FLC, 32 μg/ml + 2 μg/ml or 32 μg/ml + 256 μg/ml BBR/FLC and the same amount of DMSO were added to the yeast cells and incubated at 37°C for 72 h. The suspension was washed three times with phosphate buffered saline (PBS). The cells were then fixed with 3% glutaraldehyde and 1% osmium acid solution. Cells were then dehydrated with a series of different grades of ethanol before embedding and ultrathin sections were prepared and observed in a transmission electron microscope (Japan, HTACHI company, type H-7650).

### Measurement of intracellular reactive oxygen species

The cell suspension was treated with drugs as in the previous experiment and the same volume of DMSO was used as control. Incubate the cells for 24 h at 37°C, 7500 rpm for 5 min, resuspend them in sterile PBS, adjust the concentration to 6 × 10^6^ CFU/ml, add DHR-123 (dihydrorhodamine) to increase the final concentration to 5 μg/ml, and place them at 37°C to avoid light. Incubate them for 30 min, measure the fluorescence intensity with a flow cytometer and determine the ROS content in the cells.

### HPLC evaluation

As described in the literature (Shao et al., 2016) 5 × 10^6^ CFU/ml cells were collected and treated with drugs, and the same amount of DMSO was used as control. After 24 h of shaking culture at 37°C, ergosterol was extracted: each group weighed 0.5 g, added saponifier-containing ethanol solution and was placed in a 90°C water bath for 120 min, shaken every 30 min, 2 ml of absolute ethanol was added and extracted, and finally the upper solution was filtered with a 0.22 μm microporous membrane. The ergosterol standard product was taken at a storage concentration of 20 mg/ml. Then a solution ranging from 0.0015–1.2 mg/ml was prepared with absolute ethanol and a standard curve was constructed by linear regression. The chromatographic column is an XTerraRMS C18 (4.6 mm × 250 mm 5 μm), the mobile phase is 100% methanol, the flow rate is 1.0 ml/min, and the detection wavelength is 284 nm. Injection volume: 20 μl; detection sensitivity: 0.01 AUFS. The upper solution of each group was detected by chromatography, and the content of ergosterol in each group of fungi treated with different drugs was calculated using the linear relationship.

### Real-time PCR

One hundred milliliters of 5 × 10^3^ cells were collected and 100 ml of different concentrations of drugs were added respectively, and the concentration of DMSO in the solution was not higher than 0.5%. The cells were then incubated for 48 h with shaking in a constant temperature shaker at 37°C. The cells were then collected to extract the RNA: 200 μl of pre-cooled chloroform was added, mixed gently and left at room temperature for 5 min and then was centrifuged for 15 min at 12000 rpm in a 4°C low temperature centrifuge. 400 μl of the upper water phase was then removed and placed in a clean microtube. Then 500 μl of pre-cooled isopropanol was added, mixed gently and allowed to left stand at room temperature for 10 min. Centrifuge at 12,000 rpm for 10 min in a low temperature centrifuge at 4°C. Used Nanodrop2000 (Thermo Scientific, Waltham, MA) to detect the RNA concentration and verify the stability by a gel running. Real-time quantitative PCR (qRT-PCR) measured the mRNA expression of *ABC1*, *AtrF*, *Mdr1*, *MFS*, *PMFCZ*, and *Cyp51B*. Primer primer5 was used to design primers, and the target fragment was amplified between 100–200 bp (Table 1). The feasibility of the primers was verified by PCR. In each group, 3 parallel wells were established. A fluorescent quantitative real-time PCR instrument (BIO -RADCFX96) was used for amplification, and the cDNA loading error was adjusted by the internal reference gene *β-actin*. The reaction system was performed according to the instructions of SYBR Premix Ex TaqTMII (TiiRNaseHPlus). And with thermal cycling as follows: initial step at 95°C for 60s, followed by 40 cycles at 95°C for 15 s, 55°C for 15 s, and 72°C for 45 s. Relative fold changes of the gene were calculated using the formula 2^−ΔΔCt^.

**Table 1:** Primer sequences of gene *β-Actin*, *ABC1*, *AtrF*, *MFS*, *MDR1*, and *PMFCZ* and *Cyp51B.*

Primer name	Sequence
*Β-Actin*	(F)5′- A C G C T C C T G C C T T C T A T G T C-3′
	(R)5′ - A A C A C G G G A G A T A G C G T G A G-′
*ABC1*	(F)5′ - G A T T C G C T C C G T T A C T T C T T T C G-3′
	(R)5′- C C T C C T T T G A C A T C C A C C T C G-3′
*AtrF*	(F)5′ - T G A T T T C C C A T T C C T C G C T A C-3′
	(R)5′ - G C C T G C G T C A A C A T C C A A-3′
*MDR1*	(F) 5′ - T G G C G A G C G A G G T T T C T T-3′
	(R) 5′ - A T G T G C C G T T T T G A T T G T G G-3′
*MFS*	(F) 5′ - A C T G G C T C T C A A A T C C C A A T C T A C-3′
	(R) 5′ - C A G A A C A A A C C A A A T C C A A C G A-3′
*PMFCZ*	(F) 5′ - T G G T T G C T A C G A A G T C C A A C T-3′
	(R)5′ - T C A C A A A G A A C C T T C C A A T G C-3′
*Cyp51B*	(F)5′ - G G T G T T C C A G C A A C T G A T T A C T C T T-3′
	(R)5′ - C G C A T C C C T T C T C T C G T A T T G - 3′

### Statistical analysis

All experiments are performed in triplicate. Experimental results are all expressed as mean ± standard deviation, calculated using SPSS22.0 statistical software and statistically analyzed by one-way analysis of variance. *p* < 0.05 indicates that the difference is statistically significant.

## Results

### *In vitro* antifungal activities

As already shown in a present study, our results confirmed that for WT, the MICs of FLC and BBR alone were 2 and 32 μg/ml, respectively, and the MICs of the combination were 0.5 μg/ml of FLC and 8 μg/ml of BBR, with a FICI of 0.5 (synergistic effect). For B4 strain, the MIC of BBR was also 32 μg/ml, while the MIC of FLC was 256 μg/ml. However, the MICs of both drugs decreased to 8 μg/ml after combination and reached a FICI of 0.28, indicating a synergistic effect (FICI ≤0.5; Table 2).

**Table 2:** Susceptibility activities of BBR and FLC alone and in combination against *T. marneffei.*

Isolates	Alone MIC(μg/ml)			Combination MIC(μg/ml)		FICI	Mode of interaction
	BBR	FLC		BBR	FLC		
WT	32	2		8	0.5	0.5	Synergism
B4	32	256		8	8	0.28	Synergism

FICI: fractional inhibitory concentration index; synergy, FICI ≤ 0.5; indifferent, FICI>0.5 and ≤ 4.0; antagonism, FICI>4.0.

### Growth curve

**Figure 1:**
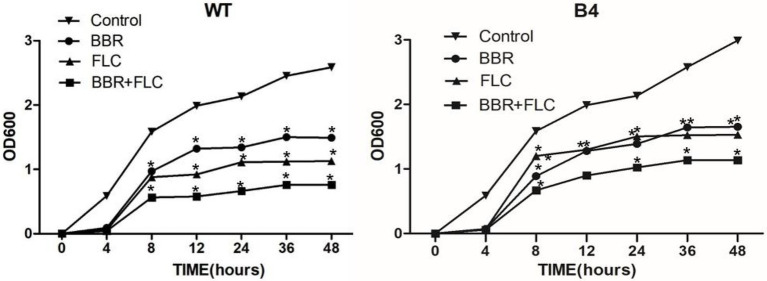
**(WT)**. Growth curve of *T. marneffei* type strain FRR 2161 treated with BBR and/or FLC Growth curves of the synergism of BBR with FLC againast *T. marneffei* type strain FRR 2161 that were obtained by using initial inoculums of 10^3^ CFU/ml. BBR (32 μg/ml); FLC (2 μg/ml); BBR (32 μg/ml) + FLC (2 μg/ml). **(B4)**. Growth curve of **(B4)** treated with BBR and/or FLC. BBR (32 μg/ml); FLC (256 μg/ml); BBR (32 μg/ml) + FLC (256 μg/ml). *p < 0.05 compared with the control.

The growth of the WT strains treated with 32 μg/ml BBR alone with 2 μg/ml FLC was significantly slower than that of untreated strains, whereas growth of strains treated with the combination (8 μg/ml BBR and 0.5 μg/ml FLC) was the significantly inhibited (*p* < 0.05; Figure 1-WT). Similarly, BBR (32 μg/ml)/FLC (256 μg/ml) alone or in combination (8 μg/ml BBR and 8 μg/ml FLC) had a similar effect to B4 strain (*p* < 0.05; Figure 1-B4).

### Transmission electron microscopy

**Figure 2:**
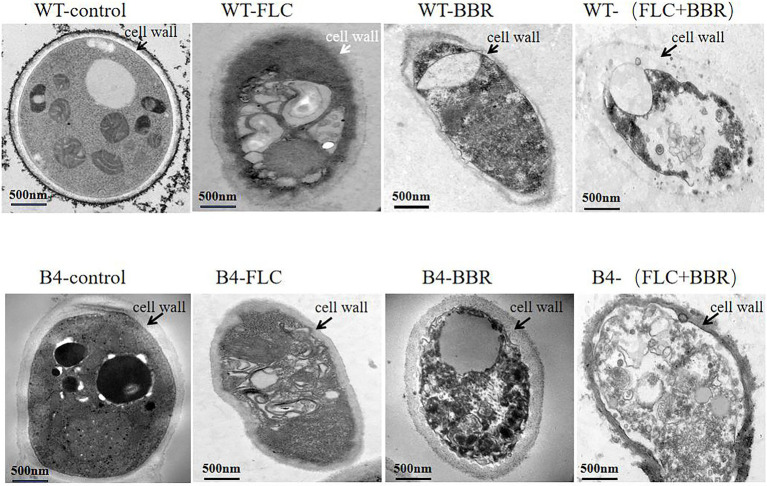
Transmission electron microscopy observation of cells **(WT,B4)** before and after using FLC or/and BBR. For **(WT)**, add BBR 32 μg/ml, FLC 2 μg/ml, BBR/FLC 32 μg/ml + 2 μg/ml; For **(B4)**, add BBR 32 μg/ml, FLC 256 μg/ml, BBR/FLC 32 μg/ml+ 256 μg/ml.

Transmission electron microscopy showed that the WT and B4 cells showed a classical morpholohy. The unique difference between these two strains was that cell wall of B4 was thicker than that of WT. The cells of WT and B4 were most severely damaged by the combined effect of the two drugs, showing incomplete cell wall and cell membrane and lysis of organelles (Figure 2).

### ROS

**Figure 3:**
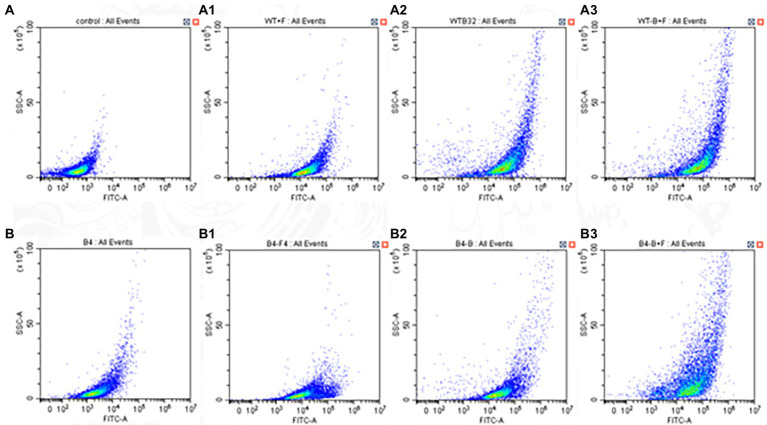
Effects of BBR and/or FLC on ROS production in **(WT,B4)**. For **(WT)**: Cells were treated with BBR (32 μg/ml) and/or FLC (2 μg/ml) 24 h and incubated with DHR-123 for 30 min. For **(B4)**: Cells were treated with BBR (32 μg/ml) and/or FLC (256 μg/ml) 24 h and incubated with DHR-123 for 30 min.

The ROS-specific dye DHR-123 could be oxidized to the fluorescent rhodamine 123 by the intracellular ROS that could be detected by fluorescent microscope. As shown, the intracellular ROS of WT and B4 strain increased compared with the control after BBR and FLC alone. The intracellular ROS of the WT and B4 increased were more obvious compared with the control after the combination of FLC and BBR (*p <* 0.05; Figure 3).

### HPLC

**Figure 4:**
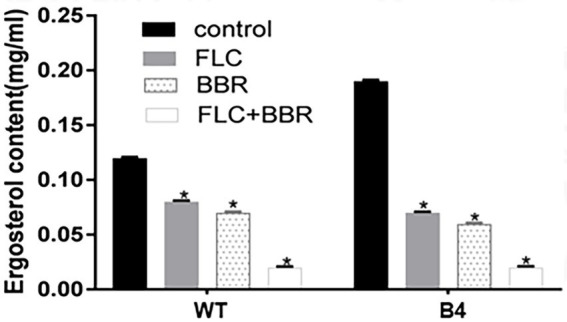
Ergosterol content surveyed by HPLC after the administrations of agents in WT and B4. *p < 0.05 compared with the control.

Compared with the control, ergosterol content in WT was reduced by 42% after using BBR, by 33% after FLC, and by 83% after BBR + FLC. Similarly, the ergocalciferol content of B4 was reduced by 69% after BBR, 63% after FLC, and 90% after BBR + FLC (Figures 4; *p* < 0.05).

### RT-PCR

**Figure 5:**
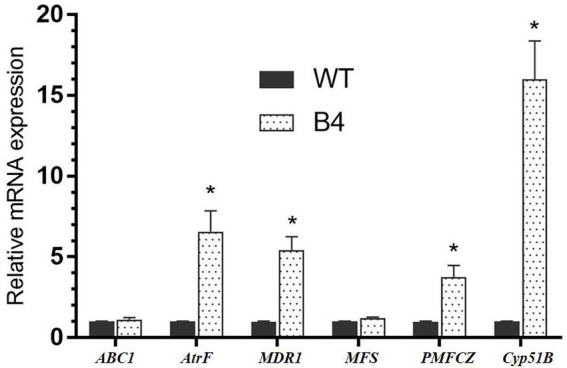
Gene expressions of important drug-related genes in WT and B4. *p < 0.05 compared with the control.

In absence of drug, the relative expression levels of the drug resistance genes *AtrF*, *MDR1*, and *PMFCZ* mRNA levels of B4 were 6.57, 5.4, and 3.76 times than that of the *T. marneffei* standard strain WT, respectively (*p* < 0.05); the expression of the B4 efflux pump genes *ABC1* and *MFS* was not significantly different from that of WT. The target enzyme gene *Cyp51B* mRNA level of FLC-resistant strain B4 is 16 times higher than the relative expression of the standard strain WT (Figures 5).

After FLC application alone, the genes *AtrF*, *MDR1*, and *PMFCZ* of WT were less expressed than in the blank control group (*p* < 0.05). The target enzyme gene *Cyp51B* was lower than in the control but showed no significant difference (*p* > 0.05). After exposure to BBR alone, the expression of the genes *AtrF*, *Mdr1*, *PMFCZ*, and the target enzyme gene *Cyp51B* were lower than in the control group (*p* < 0.05). While *ABC1* slightly decreased compared with the control (*p* > 0.05), MFS was slightly higher than in the blank group, but there was no significant difference (*p* > 0.05).

**Figure 6:**
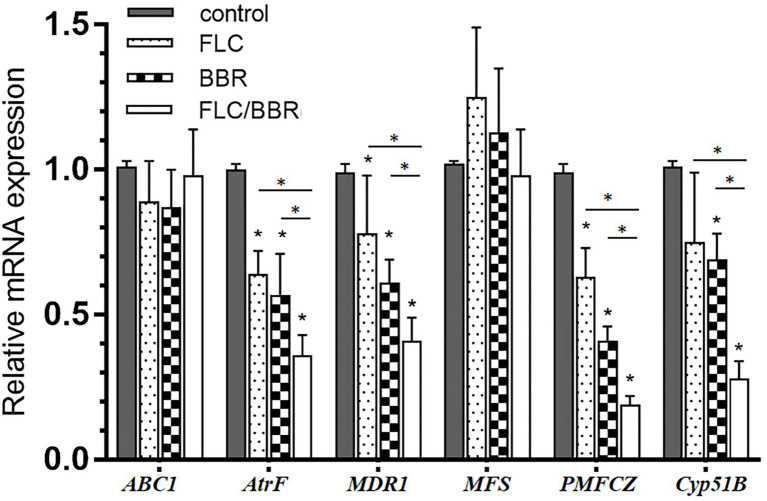
Gene expressions of *ABC1,AtrF, Mdr1,MFS, PMFCZ* and *Cyp51B* in WT under treatments. *p < 0.05 compared with the control.

**Figure 7:**
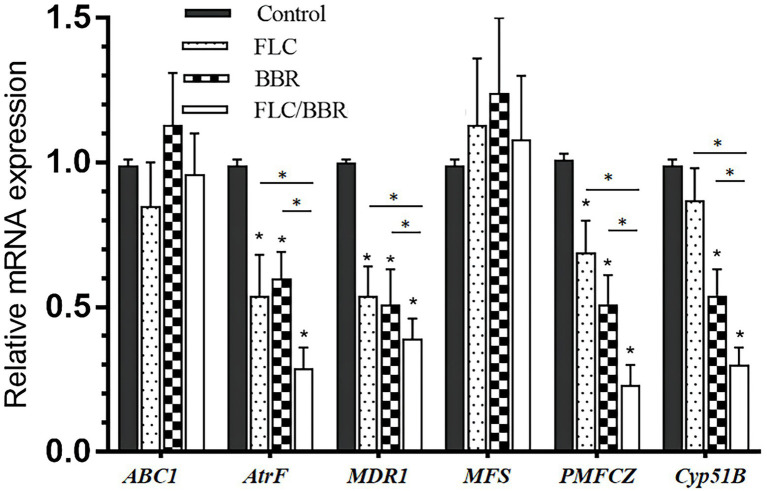
Gene expressions of *ABC1,AtrF, Mdr1,MFS, PMFCZ* and *Cyp51B* in TM (B4) under treatments. *p < 0.05 compared with the control.

After the combination of FLC and BBR, the genes *AtrF, MDR1, PMFCZ* and the target enzyme gene *Cyp51B* were expressed less than with FLC alone (*p* < 0.05), while *ABC1* was expressed slightly more than with FLC alone, but there was no significant difference (*p* > 0.05); *MFS* was slightly lower than with FLC alone, but there was no significant difference (*p* > 0.05). This shows that BBR and FLC combination has a synergistic effect to reduce the relative expression of mRNA levels of WT genes *AtrF*, *MDR1*, *PMFCZ*, and *Cyp51B* (Figures 6, 7).

## Discussion

*Talaromyces marneffei* often leads to systemic disseminated infection and high mortality. Amphotericin B is usually considered as a first choice for the treatment of *T. marneffei* infection, however, the majority of patients cannot tolerate the side effects associated to it (Zeng et al., 2015). Whereas the clinical efficacy of echinocandins in *T. marneffei* infections is poor (Nakai et al., 2003; Cao et al., 2009); the bioavailability of the oral itraconazole preparation is low (Le et al., 2017). Voriconazole, on the other hand, has good clinical efficacy and high safety in the treatment of *T. marneffei* infections, but the high cost of voriconazole is one of its disadvantages (Ouyang et al., 2017). FLC is an affordable antifungal agent with low toxicity and fewer side effects that has been used in the initial treatment of *T. marneffei* infections. However, FLC has poor clinical efficacy and a low cure rate (Supparatpinyo et al., 1993), and is not recommended in international guidelines for the treatment of *T. marneffei* infections (Masur et al., 2014).

According to the report, BBR has attracted much attention because it has very low toxicity in relatively high doses and reveals significant clinical benefits without major side effects (Chen et al., 2014), therefore, multiple functions have been explored such as anti-inflammatory, antidiabetic, antibacterial, hepatoprotective and neuroprotective effects (Chen et al., 2014; Mahmoudvand et al., 2014). In recent years, other pharmacological effects of BBR have been gradually discovered, including synergistic antibacterial effects on FLC-resistant *C. albicans*, *C. tropicalis*, and *Cryptococcus* spp, which have received increasing attention after combination with FLC (Bang et al., 2014; Chen et al., 2014; Dhamgaye et al., 2014; Shi et al., 2017). According to the literature, the mechanism of synergistic antibacterial effect of BBR in combination with FLC is mainly manifested in the following aspects: Bactericidal effect by promoting the production of intracellular reactive oxygen species (ROS; Xu et al., 2009); Acting on the expression of drug excretion pump genes, down-regulating the function of the main transposed subfamily (MFS) and ATP-binding box transporter family, and promoting intracellular drug accumulation to play a bactericidal role (Shao et al., 2016; Shi et al., 2017); Down-regulation of the expression of the gene encoding the azole drug target enzyme, wool sterol 14-α-demethylase (14DH), decreases cell membrane ergosterol synthesis, and cell membrane integrity is destroyed and susceptible to killing (Li et al., 2013). This may lead to mitochondrial dysfunction and cell death by disrupting the integrity of the fungal cell wall (Shi et al., 2017).

Our previous study found that BBR in combination with FLC had a synergistic effect on the clinical isolates of *T. marneffei in vitro* (Luo et al., 2019). Therefore, this study further investigated the mechanisms after the combined application of BBR and FLC. It was found that the combination caused significant growth inhibition and cell wall and cell membrane destruction in WT and drug-resistant strains.

BBR in combination with FLC can affect the integrity of the cell wall and cell membrane of *C. albicans* (Dhamgaye et al., 2014). It was also found that after combination, FLC-resistant *C. albicans* became sensitive to FLC by increasing the content of ROS in the cells (Xu et al., 2009; Li et al., 2013; Mahmoudvand et al., 2014). Computer aided research has revealed that BBR can embed DNA from its c5-c6-n + − C8 side (Mazzini et al., 2003). BBR can bind to double-stranded DNA and cause photooxidative DNA damage by producing ROS (Hirakawa et al., 2005), and changes its function by altering DNA structure and conformation to play an antibacterial role and induce cell apoptosis (Kumar et al., 1993), and also can be an excellent DNA intercalator rich in at sequence (Davidson et al., 1977; Iwazaki et al., 2010). In our study, in the strain WT, intracellular ROS increased after FLC/BBR alone and the combination of the two drugs. Similar results were obtained when B4 was examined. Electron microscopy examination revealed that the deformation of the nucleus and incompleteness of the cell membrane could be related to the insertion of BBR into DNA, resulting in damage to double-stranded DNA.

Up-regulation of the *Cyp51A* gene in *A. fumigatus* often leads to azole resistance (Camps et al., 2012). *Hagiwara* et al. (Hagiwara et al., 2016) recently investigated that mutations at different sites of the tandem repeats in the promoter region of the *Cyp51A* gene in *Aspergillus* can cause azole resistance. Whole genome sequencing of clinical and environmental azole-resistant strains of *A. fumigatus* in India, the Netherlands, and the United Kingdom showed that the environmental strains were mainly caused by the tr34/l98h mutation of the *Cyp51A* gene (Fan et al., 2015). In a previous study on *C. tropicalis*, significant down-regulation of *ERG11* gene expression is one of the mechanisms of the combination of BBR and FLC against *C. tropicalis* (Shi et al., 2017). However, our research found that *Cyp51B*, not *Cyp51A*, is an important gene associated with azoles in *T. marneffei* (unpublished). In this study, ergosterol synthesis from WT and B4 significantly decreased (83, 90%) and *Cyp51B* expression significantly decreased after the application of BBR in combination with FLC. It is suggested that *Cyp51B* plays an important role in the mechanism of FLC resistance.

Yuen et al. (2003) suggested that *T. marneffei* has a gene belonging to the *MFS* family (gene *PMFCZ*), which may be related to FLC resistance. When comparing the expression levels of the drug efflux transporter and ergosterol synthesis genes between the standard *T. marneffei* strain WT and B4, we found that the expression levels of the *AtrF*, *MDR1*, *PMFCZ*, and *Cyp51B* genes were higher in B4 than in WT, suggesting that the high expression of these genes may be related to the FLC resistance of the *T. marneffei*. Previous studies have shown (Shao et al., 2016) that FLC can promote the aggregation of BBR in cells and BBR impedes the normal function of *MDR1*. Fluconazole-promoted intracellular aggregation of BBR reached an effective concentration and further enhanced the antifungal activity of BBR. We speculate that BBR and FLC may promote each other, inhibit the efflux pump, and increase their concentration in cells to achieve a synergistic antifungal effect.

In conclusion, this study further investigated the mechanisms of BBR and FLC combination return to susceptibility of FLC-resistance of *T. marneffei*. Also, our study provides a new treatment option for talaromycosis. The combination could become a new efficient, safe and cost-effective regimen for the treatment of talaromycosis. However, the mechanisms of drug action is complex. In addition to the above possible mechanisms found in our study, further investigation is needed to determine whether there are other important mechanisms, and *in vivo* needs to be further verified by animal models and clinical research.

## Data availability statement

The authors acknowledge that the data presented in this study must be deposited and made publicly available in an acceptable repository, prior to publication. Frontiers cannot accept a manuscript that does not adhere to our open data policies.

## Author contributions

PK-s, LH, ZD-y, ZY-q, and AA contributed to the data collection. PK-s and LH contributed to the laboratory work. PK-S and LH wrote the manuscript. J-PL and CC-w supervised and evaluated the process of the study. All authors contributed to the article and approved the submitted version.

## Conflict of interest

The authors declare that the research was conducted in the absence of any commercial or financial relationships that could be construed as a potential conflict of interest.

## Publisher’s note

All claims expressed in this article are solely those of the authors and do not necessarily represent those of their affiliated organizations, or those of the publisher, the editors and the reviewers. Any product that may be evaluated in this article, or claim that may be made by its manufacturer, is not guaranteed or endorsed by the publisher.
